# GlycoRNA research: from unknown unknowns to known unknowns

**DOI:** 10.1093/procel/pwaf102

**Published:** 2025-11-20

**Authors:** Li Yi, Yitong Zhou, Chi Zhang, Haojie Lu, Yixuan Xie

**Affiliations:** NHC Key Laboratory of Glycoconjugates Research, Department of Biochemistry and Molecular Biology, Institutes of Biomedical Sciences and School of Basic Medical Sciences, Fudan University, Shanghai 200032, China; State Key Laboratory of Genetics and Development of Complex Phenotypes, Greater Bay Area Institute of Precision Medicine, School of Life Sciences, Fudan University, Shanghai 200032, China; NHC Key Laboratory of Glycoconjugates Research, Department of Biochemistry and Molecular Biology, Institutes of Biomedical Sciences and School of Basic Medical Sciences, Fudan University, Shanghai 200032, China; State Key Laboratory of Genetics and Development of Complex Phenotypes, Greater Bay Area Institute of Precision Medicine, School of Life Sciences, Fudan University, Shanghai 200032, China; NHC Key Laboratory of Glycoconjugates Research, Department of Biochemistry and Molecular Biology, Institutes of Biomedical Sciences and School of Basic Medical Sciences, Fudan University, Shanghai 200032, China; State Key Laboratory of Genetics and Development of Complex Phenotypes, Greater Bay Area Institute of Precision Medicine, School of Life Sciences, Fudan University, Shanghai 200032, China; NHC Key Laboratory of Glycoconjugates Research, Department of Biochemistry and Molecular Biology, Institutes of Biomedical Sciences and School of Basic Medical Sciences, Fudan University, Shanghai 200032, China; Department of Chemistry and Liver Cancer Institute, Zhongshan Hospital, Fudan University, Shanghai 200032, China; NHC Key Laboratory of Glycoconjugates Research, Department of Biochemistry and Molecular Biology, Institutes of Biomedical Sciences and School of Basic Medical Sciences, Fudan University, Shanghai 200032, China; State Key Laboratory of Genetics and Development of Complex Phenotypes, Greater Bay Area Institute of Precision Medicine, School of Life Sciences, Fudan University, Shanghai 200032, China

**Keywords:** glycoRNA, RNA modification, mass spectrometry

## Abstract

Recent discoveries have revealed the existence of glycosylated RNAs (glycoRNA), in which glycans are covalently linked to small non‑coding RNAs and are predominantly localized to the cell surface. Since the initial discovery in 2021, glycoRNA has become an emerging field: 4 years in glycoRNA research have produced advances in labeling, imaging, and mass spectrometry that now highlight the role of glycoRNA in cell communication, immune regulation, and disease progression. In this review, we summarize current knowledge of glycoRNA biogenesis, detection techniques, and biological functions, and discuss how these findings reshape the future interface between glycobiology and RNA biology.

## Introduction

More than 170 diverse types of post-transcriptional biochemical modifications have been characterized across both coding and non-coding RNAs, especially for transfer RNAs (tRNA) and ribosomal RNAs (rRNA) (Cappannini et al., 2024; Lewis et al., 2017). These modifications confer new structures and functions by altering hydrophobicity and base pairing, thereby determining RNA fates *via* regulating gene expression and cellular phenotypes (Frye et al., 2018). Take the most common modification, N6 methyladenosine (m6A), as an example; it regulates alternative splicing, stability, and translation of mRNAs under the dynamic action of writing proteins, erasing proteins, and reading proteins (Jiang et al., 2021). Another example, 5-methylcytidine (m5C), enhances RNA stability by preventing transcript degradation and affecting subcellular localization (Trixl and Lusser, 2019). Notably, pseudouridine (Ψ) is generally considered the earliest discovered RNA modification. Its engineering into *in vitro* transcribed mRNA reduces innate immune stimulation and enhances protein expression, promoting the successful application of mRNA vaccines (Cerneckis et al., 2022; Zhang et al., 2025). Meanwhile, the targeted delivery of therapeutic RNAs to hepatocytes relies on GalNAc (N-acetylgalactosamine) modification, a glycosylation-based approach that facilitates receptor-mediated uptake (Huang, 2017; Janas et al., 2018).

**Figure 1.**
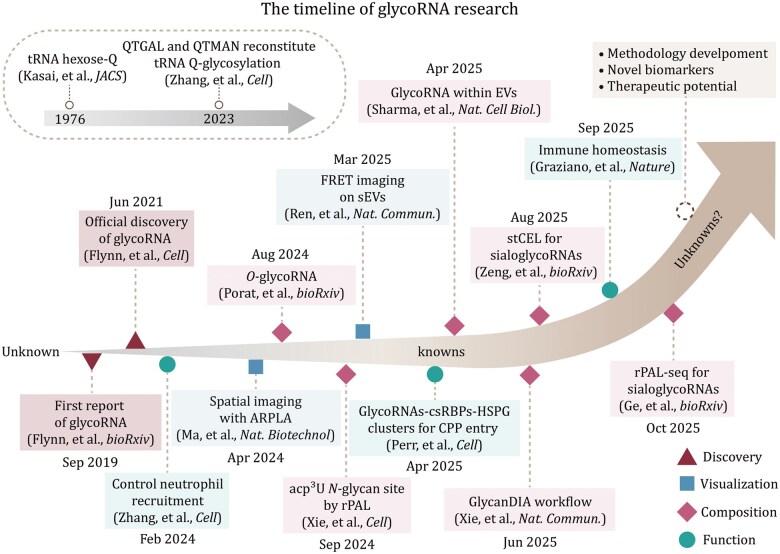
**Timeline in glycoRNA research from early hints to current advances**. Color‑coded by discovery, visualization, composition, and function.

Glycosylation is one of the most important and common modifications for proteins and lipids, in which glycans are enzymatically attached to biomolecules by transferases. Classical glycosylation is represented by *N*-linked and *O*-linked glycans, which play essential roles in protein folding, stability, signaling, and immune regulation (He et al., 2024; Reily et al., 2019). Although glycosylation of proteins and lipids has been well characterized, a link to RNA was long dismissed because RNA is confined to the nucleus or cytoplasm, whereas glycosylation occurs on the extracellular surface of the plasma membrane, with minimal spatial overlap. Nevertheless, as early as 1976, Kasai et al. reported the presence of a hexose residue on the hypermodified nucleoside queuosine (Q) in rabbit liver tRNA, providing the first hint that RNA might be modified with glycans (Kasai et al., 1976; Zhao et al., 2023). A subsequent study further showed that RNA could be capped with sugar-linked nucleotide modifications, such as uridine diphosphate glucose (UDP-Glc) and uridine diphosphate *N-*acetylglucosamine (UDP-GlcNAc), thereby extending the paradigm of post-transcriptional regulation beyond the canonical 7-methylguanosine cap (Wang et al., 2019; Weber et al., 2024). However, due to limitations in analytical tools and a lack of mechanistic insight, these observations did not gain substantial traction until 2021, when Flynn et al. reported a pioneering study challenging this view and providing evidence that mammals use RNA as a third scaffold for glycosylation (Flynn et al., 2021). This novel discovery was made possible by advances in bioorthogonal chemistry, high-throughput sequencing, mass spectrometry (MS), and high-resolution imaging, which collectively enabled the identification of RNA glycosylation and the investigation of its biological functions (Fig. 1).

**Figure 2.**
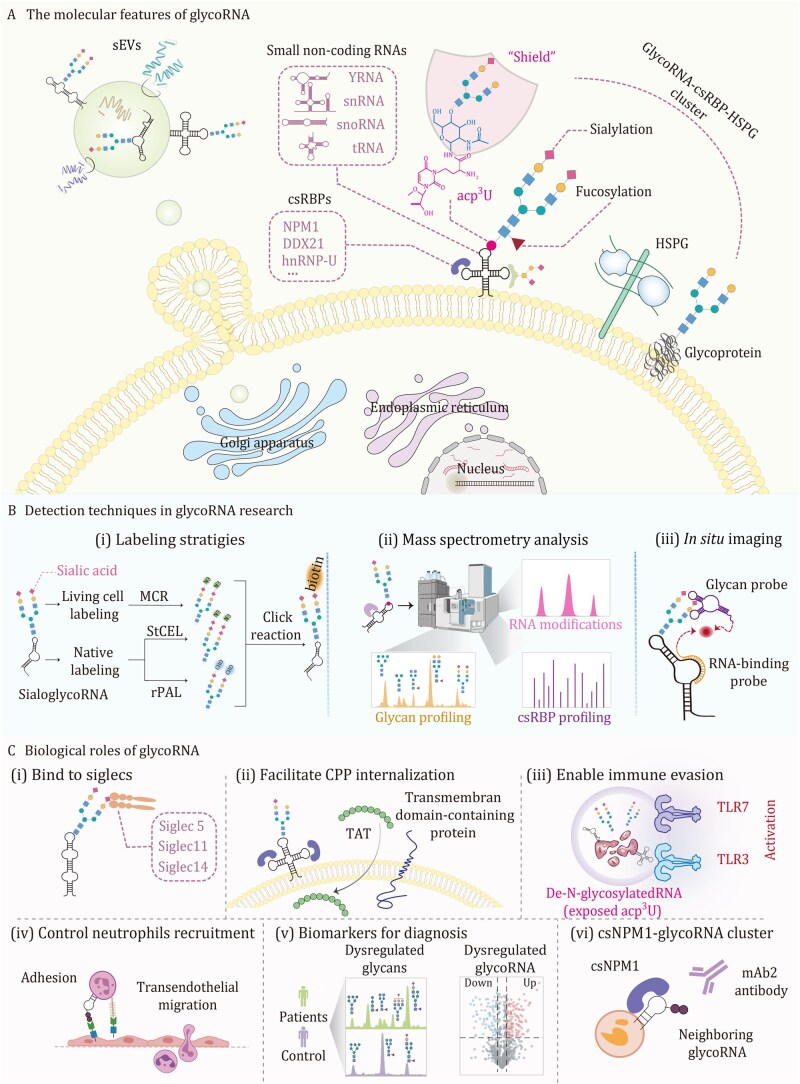
**An overview of molecular features, detection techniques, and biological roles for glycoRNA**. (A) Molecular features of ­glycoRNA. GlycoRNA localizes to the cell surface and to small extracellular vesicles, both within vesicular cargo and on vesicle membranes. They are enriched in small non-coding RNAs (Y RNA, snRNA, snoRNA, tRNA) that carry sialylated and fucosylated glycans acp^3^U serves as an *N-*glycan attachment site. At the cell surface, glycoRNA co-assembles with csRBPs, including NPM1, DDX21, and hnRNP-U, together with heparan sulfate proteoglycans (HSPG), to form nanoscale clusters. Biogenesis depends on canonical protein glycosylation machinery and ER–Golgi trafficking. (B) Detection techniques. (i) Labeling strategies: metabolic precursor labeling of sialic acids in live cells followed by click conjugation to biotin *in situ* detection with StCEL, which uses a *CstII* (sialyltransferase) to selectively transfer CMP-Neu5Az onto glycoRNA *in situ* detection with rPAL, which applies mild periodate oxidation of terminal sialic acid vicinal diols to generate aldehydes. (ii) Mass spectrometry: glycan profiling of composition and structure, identification of RNA base modifications, and profiling of csRBPs. (iii) *In situ* imaging: dual recognition with a glycan probe and an RNA-targeting probe enables visualization and quantification of glycoRNA. (C) Biological roles of glycoRNA. (i) Bind to Siglecs: sialylated glycans on glycoRNA engage Siglec receptors (e.g., Siglec5, Siglec11, Siglec14). (ii) Facilitate CPP internalization: GlycoRNA-csRBPs clusters facilitate entry of the cell-penetrating peptide TAT. (iii) Enable immune evasion: removal of *N-*glycans exposes acp^3^U on RNA and activates endosomal TLR3/TLR7, whereas intact glycans maintain immune silence. (iv) Control neutrophil recruitment: surface glycoRNA contributes to endothelial adhesion and subsequent trans-endothelial migration. (v) Biomarkers for diagnosis: dysregulated glycans and differentially expressed glycoRNA are diagnostic markers that distinguish patients from controls. (vi) csNPM1-glycoRNA clusters as targets: tumor-associated clusters comprising cell-surface NPM1 and neighboring glycoRNA can be recognized by monoclonal antibodies for selective intervention.

In this review, we provide a comprehensive overview of glycoRNA as a newly recognized class of RNA modifications (Fig. 2). First, we outline the discovery, molecular features, and biogenesis of glycoRNA. Then, we highlight key methodological advances, including metabolic labeling, mass spectrometry, and *in situ* imaging, that have enabled sensitive detection and structural characterization. We also discuss significant biological functions of glycoRNA in intercellular communication, immune regulation, and disease pathogenesis, illustrating how glycoRNA extends the functional repertoire of RNA modifications and opens new directions for RNA biology and glycobiology. For further insight into glycoRNA, readers are referred to the following reviews on extracellular and cell-surface RNA and on glycoRNA: (i) a review about types of extracellular RNAs, biogenesis, and its regulation (Chai et al., 2023); (ii) the development of proximity labeling technologies applied in RNA biology and cell surface biology (Kageler et al., 2024); (iii) a systematic overview of the cell surface RNA and RBPs, as well as their established biological roles (Porat and Flynn, 2025).

## Identification of glycoRNA

### The initial discovery of glycoRNA

Metabolic labeling of carbohydrates and bioorthogonal chemistry have been extensively used to study glycosylation (Sletten and Bertozzi, 2009). Indeed, the initial evidence of glycoRNA came from metabolic labeling with Ac_4_ManNAz (*N-*azidoacetylmannosamine-tetraacylated). Time-dependent azide signals were observed on purified RNAs after stringent removal of proteins using proteases. Notably, these signals were consistently detected across diverse cell lines and tissues, including HeLa cells, human embryonic stem cells (H9), a human myelogenous leukemia cell line (K562), a human lymphoblastoid cell line (GM12878), a mouse T cell acute lymphoblastic leukemia cell line (T-ALL 4188), and Chinese hamster ovary cells (CHO). These results suggested an unexpected and conserved link between RNAs and sialylated glycans. Further experiments confirmed that these molecules were RNAs, as they were degraded by RNase but remained resistant to DNase. The presence of sialic acids on glycoRNA was then demonstrated using two complementary approaches. First, treatment with sialic acid-specific enzymes and inhibitors, including *Vibrio cholerae* sialidase (VC-Sia) and peracetylated 3-fluoro-3-deoxy-*N-*acetylneuraminic acid (P-3Fax-Neu5Ac), led to a marked reduction in glycoRNA signals, indicating the role of sialic acid in their composition. VC-Sia hydrolyzes terminal sialic acids, whereas P-3Fax-Neu5Ac blocks their *de novo* synthesis. Second, independent of metabolic labeling, free sialic acids from purified RNAs were derivatized with the fluorogenic 1,2-diamino-4,5-methylenedioxybenzene (DMB) probe and analyzed by high-performance liquid chromatography with fluorescence detection, which identified *N-*acetylneuraminic acid (Neu5Ac) and *N-*glycolylneuraminic acid (Neu5Gc). Importantly, both forms were sensitive to VC-Sia and RNase pretreatment, further confirming the sialylated glycoRNA. Collectively, these results provide preliminary evidence for the presence of sialoglycoRNA among different cell lines.

### GlycoRNA maps to small non-coding RNA sequences

Having established the preliminary glycoRNA identity, what remains unclear are their molecular components and the types of RNA that are modified. First, Poly-A enrichment failed to capture glycoRNA, excluding them as long polyadenylated transcripts. Length-based fractionation was then used to separate RNAs into >200 nt and <200 nt populations, and glycoRNA reproducibly segregated with the small RNA fraction (<200 nt). These results were further validated using sucrose gradient separation, where glycoRNA again fractionated with small RNAs. Despite their small size, glycoRNA migrated unusually slowly in denaturing agarose gels and sucrose gradients, likely due to their associated glycans. Subsequently, Ac_4_ManNAz-labeled small RNAs enriched from gradient fractions were subjected to RNA sequencing, which revealed the presence of diverse RNA types, including small nuclear RNAs (snRNAs), small nucleolar RNAs (snoRNAs), tRNAs, and microRNAs (miRNAs). Indeed, knockout of Y RNAs (one type of snRNAs) by CRISPR-Cas9 markedly reduced the Ac_4_ManNAz signal, confirming Y RNAs as major contributors to the glycoRNA pool (Flynn et al., 2021). Consistent enrichment patterns reported in other studies support the conclusion that glycoRNA map to a broad repertoire of small RNA species (Deng et al., 2025; Hazemi et al., 2025; Ren et al., 2025; Zeng et al., 2025).

### GlycoRNA biogenesis depends on canonical N-glycosylation pathways

Canonical *N-*glycosylation begins in the endoplasmic reticulum (ER), where a glycan precursor is added to the protein and partially trimmed. The process then continues in the Golgi apparatus, where the glycans undergo further modifications to form fucosylated and sialylated structures (Bourne and Henrissat, 2001). To explore if glycoRNA biogenesis follows these canonical pathways, a series of experiments were conducted, and demonstrated that glycoRNA biogenesis indeed depends on UDP-Gal/UDP-GalNAc metabolism. In *ldlD* CHO cells, which cannot produce UDP-Gal (required for *N-*glycan elongation) and UDP-GalNAc (required to initiate *O-*glycosylation), glycoRNA signals were almost completely lost in minimal media but could be restored by galactose supplementation. A similar result was observed in the human K562 cell line with a CRISPR-Cas9 targeted knockout of UDP-galactose-4-epimerase (GALE), which mimics the phenotype of the *ldlD* CHO cell line (Flynn et al., 2021). Consistent findings were also reported in the study about exosomal glycoRNA by Kiledjian and co-workers (Sharma et al., 2025). They proved that knockdown of GALE or UDP-mannose-4-epimerase (GNE) disrupted UDP-GalNAc interconversion, leading to precursor accumulation and enhanced incorporation of the metabolic probe Ac_4_GalNAz.

The impact of canonical transfer/maturation steps on glycoRNA biogenesis was also evaluated. Inhibition of the oligosaccharyltransferase (OST) complex by NGI-1, which transfers *N-*glycans to nascent polypeptides in the ER, caused a dose-dependent loss of glycoRNA labeling (Flynn et al., 2021). Among catalytic subunits of OST, STT3A-dependent activity was particularly important for glycoRNA expression (Liu et al., 2024b). Consistently, inhibition of downstream *N-*glycans maturation in the ER and Golgi with kifunensine (α-mannosidase I inhibitor) or swainsonine (α-mannosidase II inhibitor) also led to dose-dependent reductions in glycoRNA labeling.

Moving from biogenesis to structure, glycans on RNAs are suggested to undergo protein-like assembly and exhibit similar *N-*glycan structures. Therefore, subsequent studies comprehensively investigated the structural features of glycoRNA-associated glycans. Treatment with PNGase F, which specifically cleaves *N-*linked glycans, nearly abolished glycoRNA signals, whereas the endoglycosidases Endo F2 and Endo F3, which cut within certain *N-*glycan types, only partially reduced them. To further define their composition, *N-*glycans were released from glycoRNA by PNGase F digestion and analyzed by porous graphitized carbon liquid chromatography-mass spectrometry (PGC-LC-MS), revealing enrichment in sialylation and fucosylation (Flynn et al., 2021). Together, these findings support the view that glycoRNAs are modified with complex and sialylated *N-*glycan-like structures.

Interestingly, Porat et al. demonstrated that RNAs can also carry *O-*linked glycans by applying RNA-optimized periodate oxidation and aldehyde labeling (rPAL) together with an optimized galactose oxidase (GAO) labeling workflow (Porat et al., 2024). Genetic disruption of the core *O-*glycosylation pathway, such as loss of C1GALT1 (the core-1 synthase) or its obligate chaperone COSMC, markedly reduced glycoRNA signals. Restoration by the sialyltransferase ST6GALNAC1 indicated that these *O-*glycans are sialylated. Mass spectrometry profiling further revealed diverse *O-*glycan structures, including Core-1 and Core-2 glycans with cell-type-specific patterns, as well as unexpected sulfated *O-*fucose glycans. Complementary evidence came from Yang and colleagues, who used GalNAcEXO to isolate Tn-containing *O-*glycosylated RNAs from pancreatic cancer cells and tissues, identifying hundreds of *O-*glycoRNA, among them 131 microRNAs carrying both *N-* and *O-*glycans (Li et al., 2025). These studies suggest that *O-*glycan also contributes to RNA modifications. However, extensive studies are still needed to decipher structural complexity and biogenesis.

### The modified acp3U acts as an attachment site for glycan in RNAs

**Figure 3.**
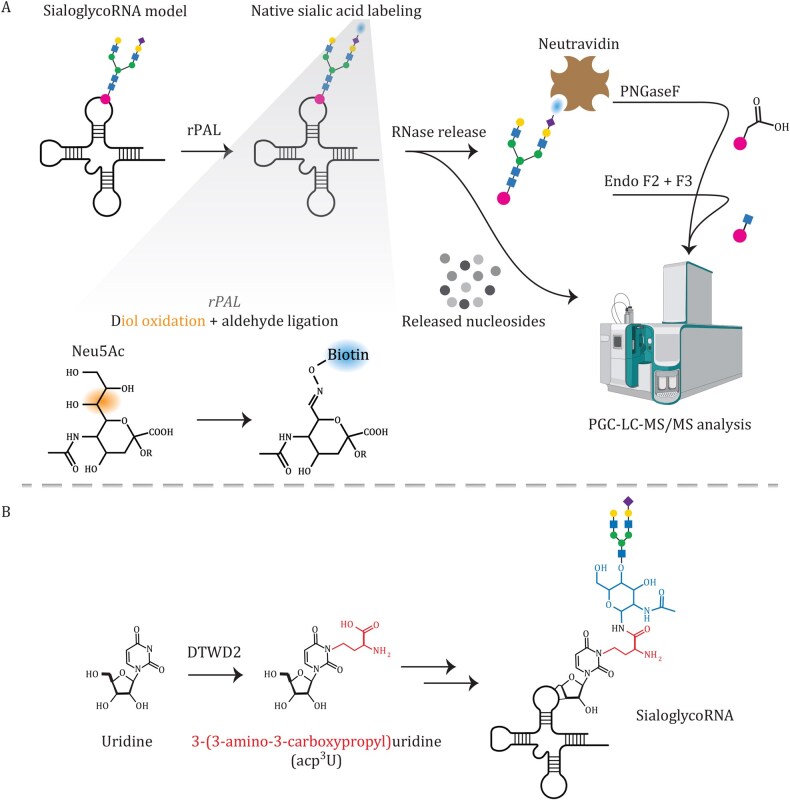
**rPAL-based native labeling and MS workflow reveal the acp^3^U *N-*glycan attachment site in glycoRNA**. (A) The rPAL selectively labels the terminal Neu5Ac of glycoRNA to aldehydes that are ligated to biotin. Labeled RNAs are digested with RNase to release nucleosides for downstream assignment, captured on neutravidin, and their N-glycans are released with PNGase F and Endo F2/F3 and profiled by PGC-LC-MS/MS. (B) DTWD2 is responsible for the synthesis of acp^3^U, and knockout of DTWD2 impacts the glycoRNA biosynthesis. Reprinted with permission from Xie et al., *Cell*, 187, 5228 (2024), copyright Elsevier (Xie et al., 2024b).

Although RNAs have been shown to carry complex *N-*glycans, direct chemical evidence of the covalent linkage between RNA nucleotides and the chitobiose core has remained elusive until recently (Disney, 2021). Establishing such a linkage has been technically challenging due to the intricate structural complexity of glycans. By developing rPAL labeling and integrating it with high-resolution MS, Xie et al. successfully enriched and characterized endogenous glyconucleosides (Xie et al., 2024). Among several candidates, the modified base 3-(3-amino-3-carboxypropyl) uridine (acp^3^U) consistently emerged across multiple enzymatic release strategies (Fig. 3). The identity of acp^3^U was validated by isotopic labeling experiments. When glycoRNA was digested with PNGase F in $H_{2}^{16}$O and $H_{2}^{18}$O, the characteristic diagnostic mass shift (+2.004 Da) was observed, indicating that acp^3^U was released from the glycan–RNA linkage *via* PNGase F. The result was further confirmed by comparison with a synthetic acp^3^U standard, which matched the chromatographic retention time and MS/MS fragmentation pattern. To find direct evidence of the linkage between acp^3^U and *N-*glycans, Endo F2/F3 digestion was employed to generate GlcNAc-modified acp^3^U. As a result, the acp^3^U-GlcNAc was detected and co-eluted with the synthetic standard and exhibited identical fragmentation spectra. In parallel, genetic evidence reinforced this conclusion, as knockout of DTWD2, which is responsible for acp^3^U biosynthesis, significantly reduced glycoRNA levels, thereby establishing a genetic basis for this modification. Together, these findings establish acp^3^U as an attachment site for *N-*glycans on RNAs, providing the first direct chemical linkage for RNA glycosylation.

### GlycoRNAs are localized on the cell surface and in small extracellular vesicles

The subcellular localization of RNA is intrinsically linked to its biological functions. For instance, nuclear RNAs can directly regulate gene expression (Statello et al., 2021), while cytoplasmic RNAs are more commonly associated with translation and degradation processes (Brito Querido et al., 2024; Schoenberg and Maquat, 2012). In the case of glycoRNA, an open question is whether glycosylation influences their localization inside cells. Subcellular fractionation was first performed to separate nuclei from the cytosol and membranous organelles. As a result, glycoRNA was not detected in the nuclear fraction. A second fractionation approach then separated the soluble cytosolic compartment from the membranous organelles. In this case, glycoRNAs were again absent from the soluble cytosol and instead exclusively recovered in the membrane fraction. To further examine their membrane topology, crude membranes and organelles were isolated and subjected to digestion with the sialidase VC-Sia, either with or without Triton X-100 to permeabilize membranes. Most glycoRNA signals were sensitive to VC-Sia without permeabilization, indicating that they are accessible on membrane surfaces. A smaller fraction became sensitive only after Triton X-100 treatment, suggesting the presence of a small glycoRNA subset within luminal compartments (Flynn et al., 2021).

To directly visualize glycoRNA on the cell surface, peroxidase-mediated proximity labeling was performed to selectively tag glycans on cells. With lectin-HRP-aniline, surface glycoRNA was detected on live HeLa cells, and the signals disappeared after RNase treatment (Flynn et al., 2021). This conclusion was reinforced by *in situ* amplification of outer membrane surface RNAs (AMOUR), a recently developed *in situ* amplification approach that enables direct imaging, qualification and sequencing of surface RNAs in living cells (Flynn, 2025; Jiang et al., 2025a). Using this method, Hu and colleagues showed that surface RNAs across multiple human and mouse blood cell types significantly overlap with glycoRNA, confirming their widespread presence on mammalian cell surfaces.

In addition to their presence on the plasma membrane, glycoRNAs have also been detected in small extracellular vesicles (sEVs). Ren et al. employed a dual-recognition Förster resonance energy transfer (drFRET) strategy combined with metabolic labeling to profile sialylated glycoRNA on the surface of HeLa-derived sEVs, providing *in situ* evidence for their stable association with the vesicle membrane (Ren et al., 2025). At the same time, Sharma et al. employed metabolic labeling in HeLa, HEK293, and MCF7 cells and showed that glycoRNAs are also sorted into the intraluminal cargo of exosomes, a subtype of sEVs, as confirmed by canonical EV markers and super-resolution fluorescence microscopy (Sharma et al., 2025).

### GlycoRNAs are broadly distributed across organisms

GlycoRNAs are widely present in humans, with signals detected in different cell lines such as HeLa, K562, H9 (Flynn et al., 2021), and immune cell populations such as monocytes (Li et al., 2025c; Ma et al., 2024), white blood cells, and red blood cells (Li et al., 2024). Interestingly, the biological roles of glycoRNA detected in enucleated RBCs remain to be elucidated. However, the glycosylation status of glycoproteins directly contributes to blood group antigen formation (Aoki, 2017). By analogy, glycoRNA in RBCs may participate in similar glycosylation-dependent mechanisms or potentially exhibit unique functions distinct from those in nucleated cells. Further studies will be required to determine their biogenesis and potential physiological relevance. GlycoRNA can also be found in bodily fluids, with strong signals in plasma and weak but reproducible levels in urine, feces, and amniotic fluid (Li et al., 2024). Moreover, in multiple mouse tissues, over 200 different *N-*glycans were identified by GlycanDIA (Xie et al., 2025). GlycoRNA from heart tissue is mainly composed of high mannose, while brain glycoRNA is mainly composed of fucose. Although sialylation structures account for more than half of glycans in most tissues, the colon and heart are rich in Neu5Ac, while the spleen contains more Neu5Gc, indicating the abundance and structural heterogeneity of glycoRNA. Besides that, Li et al. also detected glycoRNA in *Drosophila*, with only weak signals in virus and plant samples, indicating widespread evolutionary conservation (Li et al., 2024).

## Advanced detection techniques in glycoRNA research

Analyzing glycoRNA has long posed significant challenges. Their low abundance, together with the inherent instability of both glycans and RNAs, has greatly limited detection. The microheterogeneity of glycoRNA (e.g., manifested as multiple glycoforms) further complicates their identification. Recent advances in metabolic labeling, mass spectrometry, and super-resolution imaging have substantially improved sensitivity and resolution, enabling the detection of glycoRNA.

### Labeling strategies for glycoRNA detection

#### Metabolic chemical reporter

The distinguished work of Carolyn Bertozzi and colleagues in developing bioorthogonal chemistry profoundly transformed glycobiology by enabling metabolic labeling of cellular glycans (Bertozzi, 2023; Cheng et al., 2021). A landmark advance was the development of Ac_4_ManNAz, which enters the sialic acid biosynthetic pathway after cellular uptake. Once deacetylated to ManNAz, it is processed through the natural pathway to generate *N-*azidoacetyl sialic acid (SiaNAz), subsequently converted into cytidine 5′-monophosphate (CMP)-SiaNAz, and incorporated into the terminal positions of glycans on proteins and lipids (Luchansky et al., 2004). This metabolic reporter introduces an azide group that can be selectively conjugated to chemical probes via copper-free click chemistry.

Although Ac_4_ManNAz was developed nearly two decades ago (Prescher et al., 2004), its application in the discovery of glycoRNA represents a striking extension of its utility. In typical experiments, living cells are cultured with ∼100 µmol/L Ac_4_ManNAz for 24–48 h, enabling the incorporation of azido sialic acids onto RNAs. Total RNA is then extracted by TRIzol, stringently purified with proteinase K digestion to remove glycoproteins, and reacted with dibenzocyclooctyne (DBCO)-biotin under denaturing conditions. This copper-free click reaction allows the efficient and selective tagging of glycoRNA. The resulting biotinylated RNAs are separated by denaturing gel electrophoresis and visualized by blotting, allowing for the detection of glycoRNA modified with azido sialic acids. Alternatively, 9-azido sialic acid, another sialic acid metabolic probe, can also be efficiently incorporated into the termini of glycoRNA. Unlike Ac_4_ManNAz, it is directly converted into CMP-sialic acid, thereby minimizing interference with other metabolic pathways (Kosa et al., 1993).

Beyond sialic acid analogs, other per-*O-*acetylated azide sugars have also been tested for glycoRNA labeling, including Ac_4_GlcNAz (*N-*azidoacetylglucosamine-tetraacylated), Ac_4_GalNAz (*N-*azidoacetylgalactosamine-tetraacylated), and Ac_4_FucAz (6-azidofucose) (Hazemi et al., 2025). Among them, Ac_4_GlcNAz shows poor metabolic incorporation, likely due to the limitation of the UDP-GlcNAc salvage pathway, and its further conversion to ManNAz and azido sialic acid reduces labeling specificity (Saxon et al., 2002). By contrast, Ac_4_GalNAz efficiently generates both UDP-GalNAz and UDP-GlcNAz (Boyce et al., 2011; Hang et al., 2003), resulting in robust labeling signals and broad utility in *O-*glycan studies. Ac_4_FucAz, incorporated through the fucose salvage pathway (Sawa et al., 2006), exhibits very limited labeling capacity for glycoRNA, consistent with its low metabolic efficiency.

The use of diverse metabolic labeling reporters facilitates broad detection of both *N-* and *O-*linked glycans, thereby expanding the coverage of glycoRNA. However, it should be noted that azide modifications may alter glycan metabolism and distribution (Liu et al., 2022). Moreover, metabolic labeling depends on active cellular pathways and glycosyltransferase activities, making it unsuitable for fixed tissues or primary clinical samples, and prone to variability across cell types and physiological states (Batt et al., 2017; Park et al., 2018; Xie et al., 2024c).

#### rPAL to derivatize and label native glycoRNA

Given the limitations of metabolic labeling, rPAL was developed as a direct labeling approach for native sialylated glycoRNA (Xie et al., 2024b). Unlike metabolic methods, rPAL does not rely on cellular metabolism and can be applied to samples natively by leveraging periodate oxidation. Based on the reactivity of the *cis*-diols within the sialic acid molecule, rPAL oxidizes diols to generate aldehydes that are further captured by aminooxy- or hydrazide-based probes to form stable conjugates, thereby enabling selective labeling (Zeng et al., 2009). Further improvements included systematic optimization of reaction parameters (pH, ionic strength, temperature) and the introduction of pre-blocking with free aldehydes to suppress background signals, together with mucinase digestion to improve glycan accessibility. With these improvements, rPAL supports reliable detection of sialoglycoRNA from low-input samples such as sorted human peripheral blood mononuclear cells, where metabolic labeling is not feasible. Moreover, by directly capturing aldehyde-tagged sialic acids, rPAL also facilitates the *de novo* identification of glycan–RNA linkages.

Building on the rPAL technique, Ge et al. developed rPAL-seq, a sequencing platform designed for sensitive and specific profiling of native sialoglycoRNAs (Ge et al., 2025b). The method combines specific capture and release strategy with improved small RNA library construction, featuring poly(A) extension and template-switching reverse transcription to enhance coverage of structured and modified RNAs. Additional optimizations, including PEG-mediated crowding, 3′-end blocking, and UMI-assisted quantification, greatly improve sensitivity and reproducibility. rPAL-seq requires less than 100 ng of total RNA and is compatible with diverse sample types, enabling systematic analysis of glycoRNAs from both cellular and extracellular sources.

#### Sialyltransferase-mediated chemical enzymatic labeling

**Figure 4.**
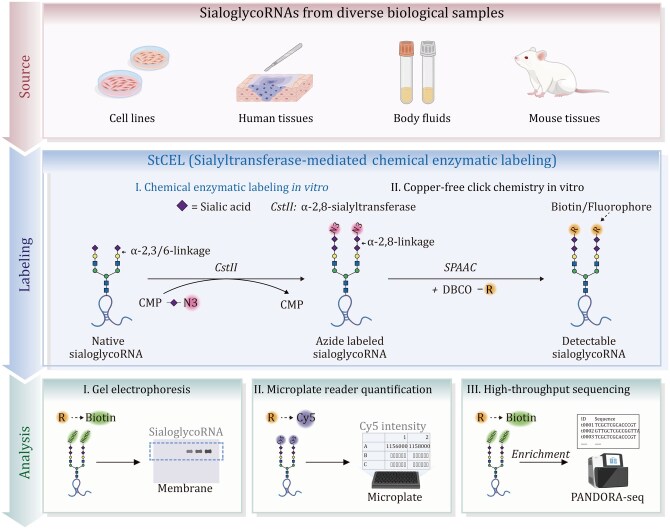
**StCEL workflow for labeling and analysis of sialoglycoRNA**. Source: sialoglycoRNA is extracted from diverse samples, including cell lines, human and mouse tissues, and body fluids. Labeling: The workflow first uses the sialyltransferase *CstII* to transfer CMP-Neu5Az onto endogenous sialoglycoRNA, followed by SPAAC conjugation with DBCO–biotin or DBCO–fluorophores. Analysis: (1) Detection: enables rapid, quantitative, and high-throughput readouts by measuring Cy5 fluorescence on a microplate reader, with optional visualization of biotin/Cy5 tags by gel or Northern blot. (2) Enrichment: streptavidin capture followed by RNA-seq provides high-throughput profiling for comprehensive characterization. Reprinted with permission from Zeng et al. (2025), bioRxiv preprint, under a CC-BY 4.0 International license (Zeng et al., 2025).

Although rPAL enables direct labeling of native glycoRNA, the potential side reaction is introduced by RNA terminal oxidation, highlighting the need for alternative strategies. To address this, Zeng et al. developed sialyltransferase-mediated chemical enzymatic labeling (StCEL), an *in vitro* strategy for native sialoglycoRNA labeling (Fig. 4) (Zeng et al., 2025). This innovative approach utilizes *CstII*, an α-2,8-sialyltransferase derived from *Campylobacter jejuni*, to selectively transfer azido-modified sialic acid (CMP-Neu5Az) to native α-2,3- or α-2,6-linked sialic acid on glycoRNA (Cheng et al., 2008; Chiu et al., 2004; Yu et al., 2009). The azido group is subsequently labeled via click chemistry with biotin or fluorescent dyes, allowing selective detection and enrichment for downstream analysis. The method proved effective in HCT116 and HeLa cells as well as in large tumor cohorts, yielding reproducible labeling signals across diverse sample types. Importantly, when combined with a microplate reader, StCEL supports high-throughput and quantitative readouts of sialoglycoRNA by measuring fluorescence signals to evaluate their abundance for potential clinical applications. Additionally, integration with PANDORA-seq enabled high-throughput sequencing (Shi et al., 2021), revealing that glycosylation is confined to specific small RNA subsets rather than being broadly distributed across whole small RNAs.

#### Galactose oxidase-based strategy

Enzymatic strategies have emerged to probe *O-*glycosylated RNAs, among which galactose oxidase (GAO) has recently been adapted for glycoRNA analysis (Porat et al., 2024). GAO is a fungal copper-containing metalloenzyme that selectively oxidizes Gal and GalNAc at the C6 hydroxyl position, generating aldehyde groups that can be chemically tagged (Whittaker, 2003). Because Gal and GalNAc are common constituents of mucin-type *O-*glycans, GAO is particularly suited for probing *O-*glycosylated RNAs. A major challenge is RNA degradation due to ribose oxidation, therefore, optimized reaction conditions were applied to minimize this problem by balancing oxidation efficiency with RNA stability (Deng et al., 2025; Li et al., 2023). GAO-based labeling not only enables enrichment of mammalian *O-*glycoRNA but also provides insights into their sialylation status, as revealed by sensitivity to sialidase treatment (Porat et al., 2024). Building on GAO-based oxidation, a solid-phase chemoenzymatic strategy was developed to enhance the specificity of *O-*glycoRNA detection (Li et al., 2025). In this workflow, oxidized RNAs are covalently immobilized on hydrazide resin, allowing stringent washing to remove nonspecific background. The immobilized RNAs are then selectively released by *O-*glycosidases (e.g., GalNAcEXO) that specifically cleave GalNAc–RNA linkages, thereby enriching Tn-containing *O-*glycoRNA for downstream analysis.

#### Lectin-guided approaches to probe glycoRNA

Lectins are glycan-binding proteins that recognize specific carbohydrate motifs with high affinity and have long been employed as tools to probe glycan structures on proteins (Weis and Drickamer, 1996; Xie et al., 2020). Their application has been extended to glycoRNA research through lectin-guided approaches. One strategy is lectin-mediated proximity labeling, which enables detection of glycoRNA on the surface of living cells (Flynn et al., 2021). In this method, biotinylated lectins bind specific glycan on the cell membrane and recruit HRP–streptavidin conjugates, which catalyze the oxidation of biotin–aniline to produce short-lived nitrene radicals that covalently tag nearby RNAs. Benchmarking in live HeLa cells confirmed robust labeling, with MAAII (sialic acid-binding) and WGA (GlcNAc ± sialic acid-binding) producing strong signals, while ConA (high mannose-specific) served as a negative control (Flynn et al., 2021).

Beyond live-cell labeling, lectins have also been adapted for blot-based detection (LBD) of glycoRNA (Li et al., 2024). In this approach, biotinylated lectins are used to directly probe total RNA. A screening of 20 lectins across THP1, HeLa, and HEK293 cells identified Lycopersicon esculentum lectin (LEL), which recognizes GlcNAc-containing glycans, as the most consistent detector of glycoRNA. Optimization of assay conditions further improved sensitivity and reduced background. Using the refined protocol, glycoRNA signals were detected not only in mammalian cells but also in diverse biological sources. However, the specificity and low-binding affinities of lectin limit its ability to detect monosaccharide-conjugated RNAs and simple glycoforms, and its application is incompatible with both fixed tissues and live-cell surfaces (Meiers et al., 2019).

### MS-based methods

**Figure 5.**
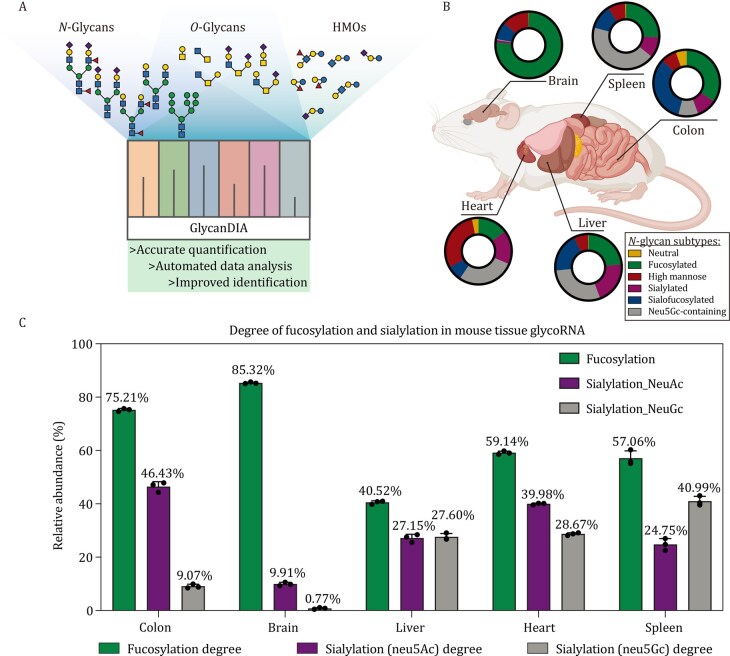
**GlycoRNA profiling by GlycanDIA across mouse tissues**. (A) Schematic of the GlycanDIA platform, which distinguishes glycan composition and isomers from *N-*glycans, *O-*glycans, and human milk oligosaccharides (HMOs), enabling accurate quantification, automated analysis, and improved identification of low-abundance modified glycans. (B) Distribution of glycan subtypes in glycoRNA isolated from different mouse tissues, shown as pie charts for brain, spleen, colon, heart, and liver. (C) Quantitative comparison of fucosylation and sialylation degrees for glycoRNA in mouse tissues. Fucosylation predominates in the colon and brain, whereas both Neu5Ac- and Neu5Gc-containing sialylation are more prominent in the liver, heart, and spleen. Values represent mean relative abundances (%). Reproduced with permission from Xie et al., *Nature Communications*, 16, 7075 (2025), copyright Springer Nature (Xie et al., 2025).

MS has greatly advanced glycobiology, providing comprehensive information on glycan composition and structural isomers with high sensitivity and resolution (Fan et al., 2025a; Ruhaak et al., 2018). Building on workflows originally established for glycoproteins, MS-based approaches have been adapted to enable direct characterization of *N-*glycans from glycoRNA. Among MS acquisition strategies, data-dependent acquisition (DDA) is widely used, in which the instrument automatically selects the most abundant precursor ions detected in a survey MS scan for subsequent fragmentation (Li et al., 2020). Typically, the “top N” precursors (e.g., top 5 or top 10 ranked by intensity) are isolated and fragmented to generate MS/MS spectra. Based on the DDA strategy, Tian and co-workers investigated glycoRNA across 12 human organs by reverse-phase LC-MS/MS (RPLC-MS/MS), and the resulting spectra were annotated using search engines such as GlySeeker and GlycoNote, yielding 676 putative glycan structures spanning 236 monosaccharide compositions (Bi et al., 2023). However, DDA is inherently biased toward high-abundance ions, resulting in inconsistent detection of low-abundance glycans. Targeted methods such as multiple reaction monitoring (MRM) overcome some of these challenges by providing high specificity and reliable quantification (Cho et al., 2022), yet they are restricted to predefined glycoforms. To overcome these limitations, data-independent acquisition (DIA, also known as “SWATH,” sequential window acquisition of all theoretical mass spectra) strategies have been introduced into glycomic analysis (Xie et al., 2025). In DIA, all precursor ions within defined m/z windows are systematically fragmented, producing comprehensive and unbiased fragmentation spectra for glycan identification (Ludwig et al., 2018). Building on this principle, the GlycanDIA workflow was developed to improve sensitivity and reproducibility (Fig. 5). It combines staggered isolation windows with higher-energy collisional dissociation (HCD)-MS/MS, while data interpretation is supported by GlycanDIA Finder, a search engine that employs iterative decoy searching for confident glycan identification and quantification. This platform enables precise discrimination of glycan compositions and isomeric structures, including *N-*glycans, *O-*glycans, and human milk oligosaccharides. Compared to traditional DDA-based workflows, GlycanDIA provides more sensitive and reproducible quantification, establishing it as a powerful and reliable tool for glycoRNA analysis.

MS is not limited to profiling glycans but can also be applied to RNA analysis (Hengesbach et al., 2025; Xie et al., 2024a). The SWAMNA (SWATH analysis of modified nucleic acids) platform was developed to support systematic characterization of RNA modifications using MS (Xie et al., 2022, 2023). It integrates enzymatic digestion with permethylation, which improves hydrophobicity and chromatographic resolution, and employs ZenoSWATH acquisition for sensitive and unbiased fragment ion coverage. Data analysis through the NuMoFinder search engine ensures robust identification and quantification. Using this strategy, acp^3^U was successfully identified as a glycan attachment site, demonstrating the potential of SWAMNA to uncover novel glycosylation linkages (Xie et al., 2024b).

Beyond the glycan and the RNA itself, RNA-binding proteins on the cell surface (csRBPs) are also essential for elucidating glycoRNA biology. MS-based proteomics characterizes RNA‑binding proteins (RBPs) with high resolution, providing both qualitative identification and quantitative protein information (Marchese et al., 2016). Furthermore, cross‑linking MS maps protein–RNA and protein–glycan interaction sites at peptide‑level resolution (Kramer et al., 2014; Xie et al., 2021). Taking advantage of it, Perr et al. leveraged proximity labeling combined with LC-MS/MS, successfully profiling multiple RBPs on the cell surface, which co-localized with glycoRNA, such as DDX21 and hnRNP-U (Perr et al., 2025).

### *In situ* imaging of glycoRNA

*In situ* detection captures biomolecules within their native cellular or tissue environments, preserving spatial information (Jia et al., 2021; Jiang et al., 2025b; Zhang et al., 2014). It relies on specific probes (e.g., hybridization probes, antibodies, or chemical tags) combined with imaging techniques. In the context of glycoRNA, this principle has been adapted into dual-recognition strategies, in which one probe targets the glycan moiety (e.g., aptamers or lectins) and another recognizes the RNA sequence. A detectable signal is generated only when both probes bind in close proximity, ensuring specificity for glycoRNA over glycans or RNAs alone. Based on this principle, Lu and co-workers developed the aptamer-based RNA-proximity ligation assay (ARPLA) (Ma et al., 2024). This method employs a proximity-driven ligation mechanism coupled with rolling circle amplification (RCA) to achieve sensitive *in situ* detection of glycoRNA. Two independent probes are required: a glycan probe consisting of a Neu5Ac-specific aptamer linked via a spacer to a DNA sequence (linker G), and an RNA probe, which hybridizes to the target RNA and carries a second DNA sequence (linker R). When both probes bind their respective targets in close proximity on the cell surface, the linker sequences are brought together and ligated into a closed circular DNA template. RCA then generates long single-stranded concatemers, which are visualized by fluorophore-labeled oligonucleotides, producing a strong fluorescent signal.

While ARPLA provides specificity, it is restricted to probing a single RNA-glycan pair at a time. To address this limitation, Liu and colleagues developed the Second-Generation Hierarchical Coding Strategy (HieCo2), which allows broader detection of glycoRNA on the cell surface (Liu et al., 2024). In HieCo2, metabolic labeling first introduces azide groups into sialic acids, which are then conjugated to a DNA “sialic acid code” (SC) via click chemistry. In parallel, an “RNA code” (RC) hybridizes with the RNA of interest. Both codes remain inactive until sequentially unlocked: the RC is initially blocked by a complementary strand (TC) that is later displaced by TC, while the SC is designed as a hairpin that only opens upon addition of a decoding primer (P). Once both codes are activated, a hybridization chain reaction (HCR) between fluorescently labeled DNA hairpins (H1 and H2) produces strong, amplified signals. This hierarchical coding ensures that only sialic acids covalently linked to the target RNA are detected. Beyond sensitive detection of low-abundance glycoRNA, HieCo2 also supports quantitative analysis and functional assays, such as blocking glycoRNA–Siglec interactions with DNA barriers.

Hybridization-based approaches often require prior knowledge of RNA sequences, which limits their application in glycoRNA detection. Yang and co-workers developed a dual-labeling strategy that bypasses sequence dependence by relying on metabolic incorporation (Ge et al., 2025). Specifically, cells are supplemented with the nucleoside analog 5-vinyluridine (5VU), which is incorporated into nascent RNAs and introduces a vinyl handle. This handle reacts selectively with tetrazine (Tz)-modified probes through an inverse electron-demand Diels-Alder (IEDDA) reaction. In parallel, Ac_4_ManAz labeling installs azide groups onto sialic acids. Because vinyl and azide chemistries are fully orthogonal, the RNA and glycan of a glycoRNA molecule can be independently targeted without cross-reactivity. On this dual-labeling foundation, the method employs a sialic acid probe (SP) and an RNA probe (RP) for selective recognition of sialylated RNAs. To minimize false positives, both probes are initially locked and only activated upon the addition of a trigger strand. Once activated, the proximity of SP and RP on the same glycoRNA induces hybridization, unfolding the RP hairpin and exposing domains that initiate a hybridization chain reaction (HCR). This cascade amplification generates strong fluorescent signals. Notably, by relying on metabolic incorporation rather than sequence-specific hybridization, this strategy detects all sialylated RNAs in a single experiment. To resolve dense molecules at the nanoscale, Fan et al. developed Molecule Differentiation Encoding Microscopy (MDEM), enabling digital and quantitative visualization of biomolecules (Fan et al., 2025). MDEM uses Orthogonal Tandem Repeat DNA Identifiers (OTRDI) to encode identical copies into different types of DNA barcodes. Coupled with proximity ligation and *in situ* RCA, MDEM was applied to visualize and quantify cell-surface glycoRNA. As a result, in single MCF-7 cells, an average of 17% copies of U1 glycoRNA were observed to gather in various nano environments on the cell surface.

The above methods rely on enzymatic or chemical signal amplification (RCA or HCR), which requires complex and time-consuming workflows. Therefore, Ren et al. developed the drFRET strategy as a simpler and faster alternative (Ren et al., 2025). Leveraging the energy transfer, drFRET accelerates one-step detection to ∼1.2 h, which is well suited for high-throughput applications. In this strategy, a glycan-recognition probe consisting of a Neu5Ac-specific aptamer labeled with a donor fluorophore (e.g., Cy3) and an RNA-targeting probe labeled with an acceptor fluorophore (e.g., Cy5) binds simultaneously to the same glycoRNA molecule. When both probes are positioned within 1–10 nm, excitation of the donor fluorophore induces efficient energy transfer to the acceptor, producing a quantifiable FRET signal. The drFRET achieves simultaneous detection of RNA and glycan epitopes, making it well suited for analyzing glycoRNA within the sEVs.

## Biological roles of glycoRNA

Chemical modifications on RNA are well known to diversify RNA structure and function, influencing processes ranging from stability to translation. Extending this principle, glycosylation represents the epitranscriptomic layer that endows RNAs with novel biological roles. Benefiting from the application of advanced analytical and detection methods, a growing number of studies are now beginning to uncover the biological roles of glycoRNA.

### Cell-surface glycoRNA functions as ligands and signaling scaffolds

The unusual localization of glycoRNA on the cell surface raises the possibility that they participate in extracellular communication. In this context, cell-surface glycoRNA may function as ligands by engaging with binding partners. To examine this, Flynn et al. first tested whether established RNA-recognition tools could detect them (Flynn et al., 2021). The J2 anti-dsRNA antibody, which selectively binds double-stranded RNA regions and is widely used to study viral RNAs (Mateer et al., 2019; Schönborn et al., 1991), was found to recognize glycoRNA, such as Y5, that contains short duplex structures. Using J2 as a probe, flow cytometry revealed glycoRNA signals on the surface of HeLa cells, with ∼20% of the population staining positive. This signal was abolished by RNase treatment and reduced by pharmacological inhibition of the OST complex, indicating that the surface display of glycoRNA depends on *N-*glycosylation. Taken together, these findings demonstrate that cell-surface glycoRNA are recognized by anti-RNA antibodies.

To explore mechanisms for the transport of glycoRNA to the plasma membrane, Ma et al. conducted imaging analysis and showed that glycoRNA co-localizes with SNARE proteins, a family of membrane fusion factors that mediate vesicle exocytosis, including TSNARE1 and VTI1b. These observations suggest that vesicle-mediated trafficking may contribute to the delivery and stable display of glycoRNA at the cell surface (Ma et al., 2024). Once delivered to the plasma membrane by vesicles, glycoRNA can act as ligands in cell–cell communication and signaling. For instance, the glycan moieties of glycoRNA can act as ligands, directly engaging specific sialic acid-binding immunoglobulin lectin-type (Siglec) members, including Siglec-11, Siglec-5, and Siglec-14 (Flynn et al., 2021; Li et al., 2025; Liu et al., 2024b). Siglecs are immune receptors that recognize sialylated glycans, and the presentation of such glycans on an RNA backbone represents a previously unrecognized mode of ligand–receptor interaction (Crocker et al., 2007). This finding highlights glycoRNA as a novel immunomodulatory ligand linking RNA biology with glycan-mediated immune signaling.

**Figure 6.**
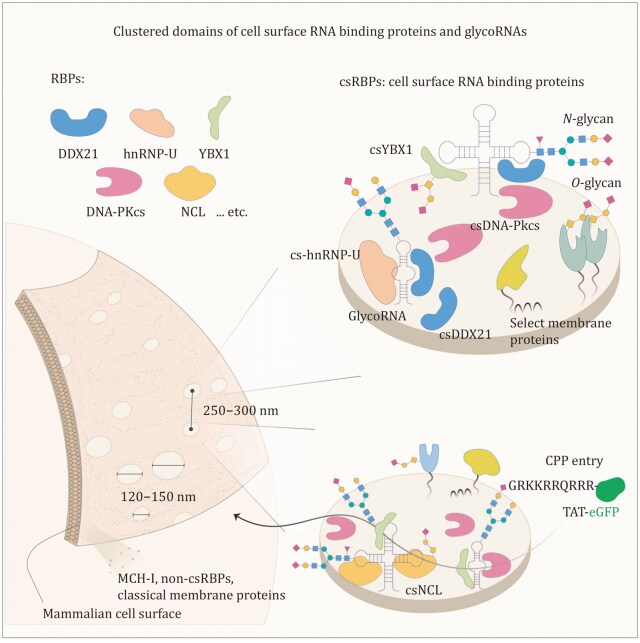
**Clustered domains of csRBPs and glycoRNA on the mammalian plasma membrane**. GlycoRNA co-localizes with csRBPs (csDDX21, cs-hnRNP-U, csYBX1, csDNA-PKcs, csNCL) to form clusters ∼120–150 nm in diameter, which are spaced ∼250–300 nm apart. These nanodomains provide functional platforms, including entry sites for cell-penetrating peptides such as TAT. Reprinted with permission from Perr et al., *Cell*, *188*, 1878 (2025), copyright Elsevier (Perr et al., 2025).

Beyond acting as individual ligands, glycoRNA assembles with csRBPs into higher-order nanoclusters (Fig. 6) (Perr et al., 2025). Super-resolution imaging revealed clusters of ∼120–165 nm that are regularly spaced at 230–300 nm, and these structures depend on intact RNA, since RNase treatment disrupts them. Functionally, these nanoclusters act as docking sites for cell-penetrating peptides (CPPs), such as HIV-1 TAT protein, offering an RNA-dependent cellular entry pathway that complements the canonical model involving heparan sulfate proteoglycans (HSPGs). Formation of these clusters requires HSPGs with extended chains carrying specific *N-* and 6-*O-*sulfation, and disruption of HSPGs or their modifications causes cluster disassembly (Chai et al., 2024). In addition to peptide uptake, glycoRNA-csRBP-HSPG clusters also regulate signaling. For instance, VEGF-A165 binds to these nanoclusters in an RNA-dependent manner. Overall, these findings highlight glycoRNA-csRBP-HSPG clusters as functional nanodomains, providing a new layer of regulation at the cell surface.

### Exosomal glycoRNA as mediators of intercellular communication

The sEVs are central mediators of intercellular communication, carrying and displaying proteins, lipids, and RNAs (Rohm et al., 2025; Thakur et al., 2022). In this context, the glycoRNA on the surface of sEVs is supposed to serve as ligands, mediating vesicle-recipient cell interactions. Indeed, their sialylated *N-*glycans, for instance, are recognized by lectin receptors like Siglec-10, Siglec-11, and P-selectin. Through these receptor–glycan interactions, glycoRNA contributes to adhesion of sEVs to recipient cells and facilitates its efficient internalization. Both the RNA backbone and its glycan modifications are essential for this process, as enzymatic removal of either markedly reduces uptake across endothelial, hepatic, and colonic cells, primarily by impairing endocytosis (Ren et al., 2025). Importantly, glycoRNAs are also selectively packaged as intraluminal cargo within exosomes, a subtype of small extracellular vesicles, in a process that appears to be tightly regulated (Sharma et al., 2025). Their export depends on canonical vesicle biogenesis pathways, including the ESCRT machinery and ceramide-associated lipid mechanisms. When either pathway is inhibited, glycoRNA accumulates inside cells and fails to be released, demonstrating that their secretion is an active and controlled process. Once secreted, exosomal glycoRNA are efficiently transferred to recipient cells and remain stable for extended periods, underscoring their role as durable mediators of intercellular RNA communication.

### GlycoRNA regulates immune tolerance

**Figure 7.**
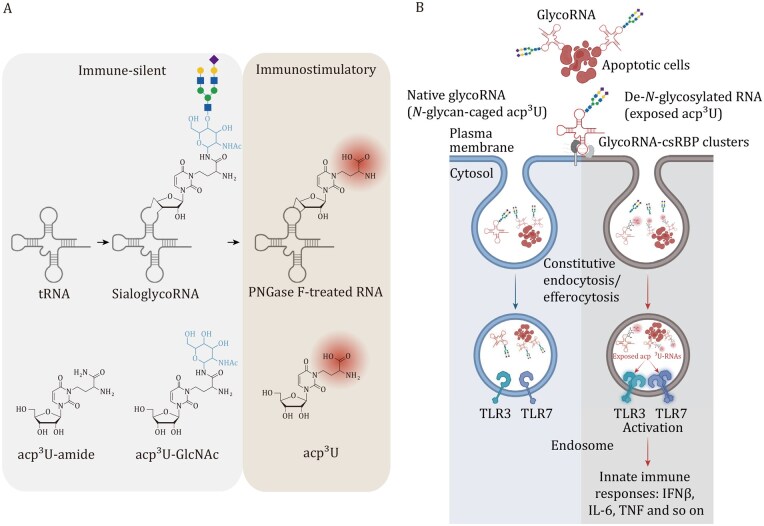
**N-glycan shielding of acp^3^U in glycoRNA gates endosomal TLR sensing**. (A) The tRNA modified at acp^3^U can be further glycosylated to form sialoglycoRNA, which remains immune-silent. Removal of N-glycans by PNGase F exposes the underlying acp^3^U, generating immunostimulatory RNA. Chemical structures of acp^3^U derivatives (acp^3^U-amide, acp^3^U-GlcNAc, and acp^3^U) are shown. (B) Native *N-*glycoRNA from apoptotic cells is internalized by constitutive endocytosis or efferocytosis and traffics into endosomes, where the *N-*glycan shields the acp^3^U modification from recognition. Upon de-*N-*glycosylation, exposed acp³U enables TLR engagement and induction of Innate immune responses (e.g., IFNβ, IL-6, TNF). Reprinted with permission from Graziano et al., *Nature*, 645, 784 (2025), copyright Springer Nature (Graziano et al., 2025).

RNA exposed on the cell surface is generally expected to trigger immune activation through recognition by endosomal sensors such as TLR3 or TLR7 (Uehata and Takeuchi, 2020). Remarkably, glycoRNAs do not elicit such responses despite their extracellular localization. This immune tolerance is conferred by their *N-*glycan modifications, which form a protective glycan shield that masks immunostimulatory RNA motifs and prevents receptor engagement. Previous studies have shown that the modified nucleoside acp^3^U can serve as an attachment site for *N-*glycans (Xie et al., 2024), making it a critical structural element in this shielding mechanism. Specifically, when glycans are enzymatically removed, acp^3^U becomes exposed and is readily recognized by TLR3 and TLR7, thereby triggering strong innate immune responses (Graziano et al., 2025) (Fig. 7). Supporting this, DTWD2-­deficient cells, which lack acp^3^U formation and thus fail to attach *N-*glycans, do not mount immune activation even after glycan removal, confirming acp^3^U as a key immunogenic epitope. Functionally, this glycan-mediated shielding is especially important during efferocytosis: glycoRNA displayed on dying cells enables macrophages to clear debris silently, whereas removal of *N-*glycans exposes acp^3^U and provokes robust macrophage activation. Thus, this glycan shield is an essential mechanism for maintaining immune silence during efferocytosis and thereby preserving homeostasis.

### Surface glycoRNA regulates immune cell trafficking

Immune cell trafficking is how leukocytes move to tissues, and it depends on their step-by-step interactions with the endothelium (Williams et al., 2011; Xia et al., 2021). Neutrophils, a type of leukocyte, respond rapidly to tissue injury by migrating from the circulation to inflammatory sites (Ley et al., 2007; Margraf et al., 2022). Unexpectedly, Lu and co-workers found the presence of glycoRNA in neutrophils, and demonstrated they engage in neutrophil endothelium interactions during inflammation (Zhang et al., 2024). In neutrophils, glycans on the RNA can be recognized by P-selectin on activated endothelial cells, supporting capture, rolling, and firm adhesion prior to trans-endothelial migration. When glycoRNAs are missing, neutrophils struggle to adhere to the endothelium and infiltrate tissues *in vivo*, showing that glycoRNAs are key players in neutrophil recruitment. Their presence at the cell surface seems to rely on the RNA transporters Sidt1 and Sidt2, which mediate RNA trafficking across membranes (Chai et al., 2023; Yang et al., 2024). Notably, blocking the glycan strongly reduces adhesion and migration, whereas inhibiting the RNA backbone has little effect, which implies glycan is the primary binding determinant. A similar glycan-dependent adhesion mechanism has been observed in human monocytes, where surface glycoRNA interacts with Siglec-5 on endothelial cells to promote adhesion (Li et al., 2025). Moreover, glycoRNA expression is dynamically regulated during immune cell differentiation and activation. In THP-1 monocytes. Treatment with phorbol esters, which drive maturation into macrophages, reduced the levels of U1, U35a, and Y5 glycoRNA. By contrast, stimulation with lipopolysaccharide (LPS), which activates innate immunity, enhances their surface expression. Removal of glycoRNA with RNase reduces immune cell-endothelial binding in multiple myeloid lineages, indicating that glycoRNA facilitates leukocyte adhesion and recruitment during inflammation.

## Clinical potential of glycoRNA

Glycosylation patterns, such as changes in sialylation, have been reported to be associated with physiological states (Cao et al., 2022; Li and Ding, 2019). Glycan-based biomarkers, especially for serum/plasma-derived *N-* and *O-*linked glycans, are already used in the clinic for diabetes and cancers (e.g., glucose/HbA1c, CA19-9, AFP-L3) (Kailemia et al., 2017; Thaçi and Anthony, 2025). RNA biomarkers from tissues and biofluids also support diagnosis and disease therapy (Delaunay et al., 2024; Shan et al., 2025). Given the ability of glycoRNA to engage cell–cell communication and immune regulation, they represent promising candidates for diagnostic and therapeutic potential.

### Diagnostic utility

**Figure 8.**
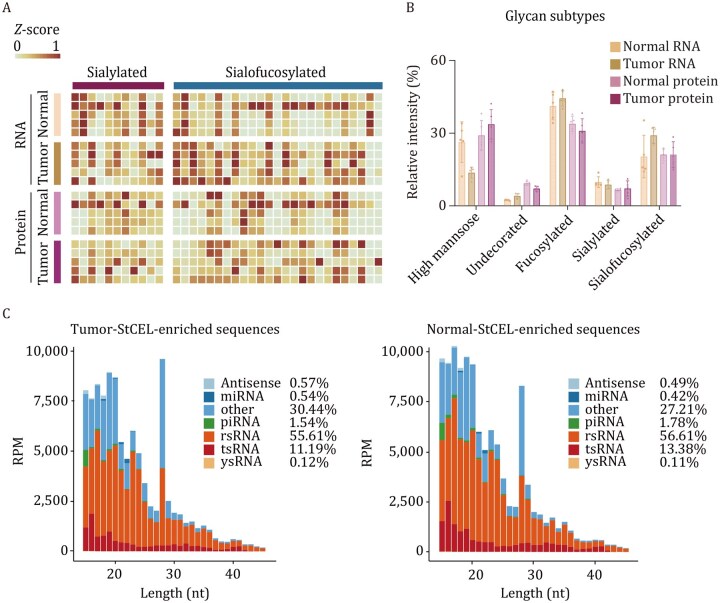
**Glycan dysregulation and differential sialoglycoRNA expression in rectal tumors**. (A) Heatmap of sialylated and sialofucosylated glycans detected from small RNAs and membrane proteins in rectal tumor and matched normal tissues. (B) Relative abundance of five glycan subtypes in glycoRNA and glycoproteins from tumor versus normal samples. (C) Length distribution and RNA types of StCEL-enriched sialoglycoRNA in five paired rectal tumors (left) and adjacent normal tissues (right). Reprinted with permission from Zeng et al. (2025), bioRxiv preprint, under a CC-BY 4.0 International license (Zeng et al., 2025).

To investigate whether glycoRNA expression is dysregulated in disease contexts. Ma et al. examined breast epithelial cell models, and the expression of glycoRNA such as U1, U35a, and Y5 was upregulated in non-malignant MCF-10A cells, while these signals declined in malignant MCF-7 and were lowest in highly metastatic MDA-MB-231 cells by quantitative ARPLA analysis (Ma et al., 2024). This inverse correlation between surface glycoRNA abundance and tumor malignancy suggests a potential tumor-suppressive role for glycoRNA in this context. Conversely, compared with adjacent normal tissues, rectal (80.5%), lung (72.5%), and esophageal (70.8%) tumors showed higher levels of sialylated glycoRNA by StCEL-based analysis (Fig. 8) (Zeng et al., 2025). Interestingly, Zeng et al. found significant enrichment of MSTRG.7832, MSTRG.5930, and MSTRG.18836 in the P3HR1, Akata, and CNE2 cell lines using the Clier-seq (click chemistry-based enrichment of glycoRNA sequencing) pipeline (Zhu et al., 2025). These results reflect different glycosylation patterns and RNA sequences of glycosylated RNAs, underscoring their potential as novel diagnostic biomarkers.

Tumor-derived sEVs are abundant and stable in the bloodstream, making them attractive targets for liquid biopsy (Li et al., 2025; Wang et al., 2025). Multiple glycoRNAs were detected on sEVs from cancer patient serum and cultured tumor cells, suggesting potential as biomarkers. Using HER2 aptamer-coated beads and the drFRET assay, researchers achieved sensitive imaging and quantification of five glycoRNAs (U1, U3, U35a, U8, and Y5) across cancer cell lines (Ren et al., 2025). Distinct glycoRNA profiles emerged depending on tissue origin, reflecting tumor heterogeneity. In clinical samples, the combined signature of these five glycoRNAs distinguished cancer patients from healthy controls and further separated cancer types, highlighting their promise for cancer diagnosis. Unsupervised clustering supported this specificity, clearly separating patient groups by glycoRNA patterns.

Beyond oncology, glycoRNAs are also associated with other pathological conditions. On alveolar epithelial cells, complex sialylated and fucosylated *N-*glycans preserve epithelial barrier integrity but simultaneously act as viral attachment sites, facilitating influenza infection (Abledu et al., 2025). In systemic lupus erythematosus (SLE), serum glycoRNA displays highly heterogeneous profiles compared with healthy individuals, suggesting disease-related dysregulation and potential as autoimmune biomarkers (Graziano et al., 2025). It has been proposed that defects in glycoRNA biogenesis may expose underlying acp^3^U nucleosides, which activate innate immune receptors and fuel autoimmune responses.

### Therapeutic potential of glycoRNA

Building on their association with disease states, glycoRNAs offer opportunities for targeted therapy. As illustrated in acute myeloid leukemia (AML) (George et al., 2025), glycoRNA assembles into nanoclusters with csRBPs such as nucleophosmin-1 (NPM1) on the cell surface in AML. It was observed that the nanoclusters localize in leukemia blasts and stem cells but are absent from normal hematopoietic stem cells. This tumor-specific signature regulated the development of monoclonal antibodies that selectively eliminate NPM1^+^ cells through immune-mediated mechanisms. In preclinical models such as patient-derived xenografts, treatment with these antibodies prolonged survival and lowered leukemia burden, with minimal effects on healthy tissues. Remarkably, this therapeutic effect extended to NPM1^+^ solid tumors, suggesting that its broadly applicable immunotherapy targets across diverse cancers.

Given the findings that glycoRNA on the cell surface plays a crucial role in recruiting immune cells to inflammation sites, Meng and co-workers leverage this feature by coating nanoparticles (NPs) with glycoRNA-rich cell membranes in abdominal aortic aneurysm (AAA) therapy (Zhang et al., 2025). Specifically, metallothionein 1 (MT1) is upregulated in aneurysm tissues and promotes the formation of neutrophil extracellular traps (NETs). However, excessive production of NETs may facilitate vascular pathological remodeling. Accordingly, glycoRNA-NP-siMT1 (a small interfering RNA against MT1) can selectively target inflamed vasculature in AAA, reduce neutrophil infiltration, and suppress NET formation, thereby slowing AAA progression, which represents glycoRNA as both regulators of vascular inflammation and a promising therapeutic strategy.

Beyond these established examples, we also highlight potential and promising therapeutic avenues. GlycoRNA-csRBPs-HSPG clusters could serve as broadly applicable targets across both hematological malignancies and solid tumors, a concept that remains to be fully validated. In addition, given the immunoregulatory roles of glycoRNA, the use of glycoRNA-coated nanoparticles to modulate inflammation represents another promising therapeutic strategy. Together, these findings underscore the emerging translational potential of glycoRNA while pointing to important “unknowns” that warrant further investigation.

## Future perspectives

Since the initial official report of glycoRNA in 2021, the field has transformed “unknown unknowns” into “known unknowns,” becoming a pivotal intersection between glycobiology and RNA biology. Indeed, such a new scientific frontier requires the traditional glycobiologist to learn the fundamentals of RNA, while RNA experts need to understand the essentials of glycobiology; such multidisciplinary expertise is crucial for advancing our understanding of glycoRNA. With years of effort, studies led by Flynn and other researchers have demonstrated the presence of sialylated and fucosylated *N-*linked glycans on small non-coding RNAs via acp^3^U, revealed their localization to the cell surface and extracellular vesicles, and uncovered their potential roles in immune regulation and disease. However, there are several key questions that remain unanswered, including (but not limited to): (i) the existence of other attachment sites of *N-*glycans and the identification of *O-*glycans attachment sites; (ii) the possibility of multiple glycosylation sites within a single RNA and whether conserved sequences exist; (iii) the enzymes and subcellular locations for glycoRNA biosynthesis; (iv) the binding proteins of glycosylated RNA; and (v) the dynamics of glycoRNA expression under physiological and pathological conditions.

Decoding these known unknowns will be challenging, while it is crucial to learn the molecular features, biological processes, and further clinical implications of glycoRNA molecules. To achieve this, the development and application of advanced technologies are fundamental. Therefore, leveraging established techniques and integrating other cutting-edge tools can provide a comprehensive view of glycoRNA. For example, RNA labeling dynamically tracks glycoRNA in cells, nanopore sequencing reveals site-specific modifications and sequences, and spatial omics maps their distribution within tissues. Combining these approaches facilitates studies of biological relevance and supports the development of glycoRNA as diagnostic and therapeutic targets.

## Abbreviations

AAA, abdominal aortic aneurysm; acp3U, 3-(3-amino-­3-carboxypropyl) uridine; Ac4FucAz, acetylated 6-azidofucose; Ac4GalNAz, acetylated N-azidoacetylgalactosamine; Ac4GlcNAz, acetylated N-azidoacetylglucosamine; Ac4ManNAz, per-O-acetylated N-azidoacetylmannosamine; AFP-L3, α-fetoprotein-Lens culinaris agglutinin-reactive fraction; AMOUR, amplification of outer membrane surface RNAs; ARPLA, aptamer-based RNA-­proximity ligation assay; C1GALT1, core 1 β1,3-galactosyltransferase; CA19-9, carbohydrate antigen 19-9; CHO, Chinese hamster ovary; Clier-seq, click chemistry-based enrichment of glycoRNA sequencing; CMP, cytidine monophosphate; CMP-Neu5Az, CMP-N-azidoacetylneuraminic acid; CPPs, cell-penetrating peptides; CRISPR, clustered regularly interspaced short palindromic repeats; CstII, campylobacter jejuni sialyltransferase; Cy3/Cy5, cyanine dyes 3 and 5; DDA, data-dependent acquisition; DBCO, dibenzocyclooctyne; DIA, data-independent acquisition; DMB, 1,2-diamino-4,5-methylenedioxybenzene; DDX21, DEAD-box helicase 21; DNase, deoxyribonuclease; drFRET, dual-recognition Förster resonance energy transfer; dsRNA, double-stranded RNA; DTWD2, DTW domain-containing protein 2; Endo F2, endoglycosidase F2; Endo F3, endoglycosidase F3; ER, endoplasmic reticulum; ESCRT, endosomal sorting complexes required for transport; EV, extracellular vesicle; FRET, Förster resonance energy transfer; GALE, UDP-galactose-4-epimerase; Gal, galactose; GalNAc, N-acetylgalactosamine; GAO, galactose oxidase; GlycanDIA, glycan data-independent acquisition workflow; Glc, glucose; GlcNAc, N-acetylglucosamine; glycoRNA, glycosylated RNAs; GM12878, human lymphoblastoid cell line GM12878; GNE, UDP-N-acetylglucosamine-2-epimerase/N-acetylmannosamine kinase; H1/H2, DNA hairpins 1 and 2; HCD, higher-energy collisional dissociation; H9, human embryonic stem cell line H9; HCR, hybridization chain reaction; HieCo2, second-generation hierarchical coding strategy; hnRNP-U, heterogeneous nuclear ribonucleoprotein U; HPLC, high-performance liquid chromatography; HRP, horseradish peroxidase; HSPGs, heparan sulfate proteoglycans; IEDDA, inverse ­electron-demand Diels-Alder reaction; K562, human myelogenous leukemia cell line K562; LC-MS, liquid chromatography mass spectrometry; LC-MS/MS, liquid chromatography tandem mass spectrometry; LBD, lectin-based detection; LEL, lycopersicon esculentum lectin; LPS, lipopolysaccharide; MCR, metabolic chemical reporter; MCF7, Michigan Cancer Foundation-7 breast cancer cell line; m5C, 5-methylcytidine; m6A, N6 methyladenosine; MDEM, molecule differentiation encoding microscopy; miRNA, microRNA; MS, mass spectrometry; MSTRG, merged transcript group; NATs, neutrophil extracellular traps; Neu5Ac, N-acetylneuraminic acid; Neu5Gc, N-glycolylneuraminic acid; NPs, nanoparticles; OST, oligosaccharyltransferase; OTRDI, orthogonal tandem repeat DNA identifiers; P, decoding primer; P-3Fax-Neu5Ac, peracetylated 3-fluoro-3-deoxy-N-acetylneuraminic acid; PEG, polyethylene glycol; PGC-LC-MS, porous graphitized carbon liquid chromatography-mass spectrometry; PNGase F, peptide-N4-(N-acetyl-β-glucosaminyl) asparagine amidase F; Q, queuosine; RBC, red blood cell; RCA, rolling circle amplification; RBPs, RNA-binding proteins; rPAL, RNA periodate oxidation and aldehyde labeling; rPAL-seq, sequencing based on rPAL; RNA, ribonucleic acid; RNA-seq, RNA sequencing; RP, RNA probe; rRNA, ribosomal RNA; sEVs, small extracellular vesicles; SC, sialic acid code; Siglec, sialic acid-binding immunoglobulin-like lectin; SiaNAz, N-azidoacetyl sialic acid; snRNA, small nuclear RNA; snoRNA, small nucleolar RNA; SP, sialic acid probe; StCEL, sialyltransferase-mediated chemical enzymatic labeling; SWAMNA, SWATH analysis of modified nucleic acids; SWATH, sequential window acquisition of all theoretical mass spectra; TAT, HIV-1 trans-activator of transcription; TLR3/7, Toll-like receptor 3/7; Tn, GalNAcα1-O-Ser/Thr antigen; tRNA, transfer RNA; TSNARE1, T-SNARE domain-containing protein 1; Tz, tetrazine; UDP-Gal, uridine diphosphate galactose; UDP-GalNAc, uridine diphosphate N-acetylgalactosamine; UDP-GalNAz, uridine diphosphate N-azidoacetylgalactosamine; UDP-Glc, uridine diphosphate glucose; UDP-GlcNAc, uridine diphosphate N-acetylglucosamine; UMI, unique molecular identifier; VC-Sia, vibrio cholerae sialidase; VEGF-A165, vascular endothelial growth factor A isoform 165; VTI1b, vesicle transport through interaction with t-SNAREs 1B; Ψ, pseudouridine; 5VU, 5-vinyluridine

## Artificial intelligence (AI)

AI-assisted tools (ChatGPT) were used for language polishing and grammar refinement during manuscript preparation.
